# Housing the Nursing Staff

**Published:** 1920-12-25

**Authors:** Henry V. Stuart

**Affiliations:** The Rectory, Stoke


### Housing the Nursing Staff.

*To the Editor of* The Hospital.

Sir,—In reference to Miss Leigh's letter last week about the nurses' accommodation at the North Staffs Cripples' Hospital, I may say that no one is satisfied with it, least of all the Committee. The evidence of this is that, in spite of an overdraft of £6,000, there is being built, as rapidly as it can be got done in these days, proper accommodation, which ought to be ready in two months' time. The present arrangements are purely temporary, caused partly by the difficulty of getting building done, but mainly by the phenomenal growth of the work; the out-patient visits increased in four years from sixty per month to 5,500.

The Committee are thankful to say that the nurses they have been able to obtain have preferred to come and work under great disadvantages, rather than cause helpless cripples to be sent away unattended to for want of nurses to care for them.—Yours faithfully,

Henry V. Stuart.

The Rectory, Stoke, December 19, 1920.

